# Equine-Like H3 Avian Influenza Viruses in Wild Birds, Chile

**DOI:** 10.3201/eid2612.202063

**Published:** 2020-12

**Authors:** Nicolas Bravo-Vasquez, Jiangwei Yao, Pedro Jimenez-Bluhm, Victoria Meliopoulos, Pamela Freiden, Bridgett Sharp, Leonardo Estrada, Amy Davis, Sean Cherry, Brandi Livingston, Angela Danner, Stacey Schultz-Cherry, Christopher Hamilton-West

**Affiliations:** University of Texas Health Science Center, Houston, Texas, USA (N. Bravo-Vasquez);; Centers for Disease Control and Prevention, Atlanta, Georgia, USA (J. Yao); University of Chile, Santiago, Chile (P. Jimenez-Bluhm, C. Hamilton-West);; St. Jude Children’s Research Hospital, Memphis, Tennessee, USA (V. Meliopoulos, P. Freiden, B. Sharp, L. Estrada, A. Davis, S. Cherry, B. Livingston, A. Danner, S. Schultz-Cherry)

**Keywords:** avian influenza, influenza, influenza viruses, viruses, equine influenza virus, wild birds, respiratory infections, surveillance, phylogenetic analysis, zoonoses, Chile

## Abstract

Since their discovery in the United States in 1963, outbreaks of infection with equine influenza virus (H3N8) have been associated with serious respiratory disease in horses worldwide. Genomic analysis suggests that equine H3 viruses are of an avian lineage, likely originating in wild birds. Equine-like internal genes have been identified in avian influenza viruses isolated from wild birds in the Southern Cone of South America. However, an equine-like H3 hemagglutinin has not been identified. We isolated 6 distinct H3 viruses from wild birds in Chile that have hemagglutinin, nucleoprotein, nonstructural protein 1, and polymerase acidic genes with high nucleotide homology to the 1963 H3N8 equine influenza virus lineage. Despite the nucleotide similarity, viruses from Chile were antigenically more closely related to avian viruses and transmitted effectively in chickens, suggesting adaptation to the avian host. These studies provide the initial demonstration that equine-like H3 hemagglutinin continues to circulate in a wild bird reservoir.

Aquatic birds are the reservoir of influenza A viruses and responsible for the evolution and long-distance spread of the virus (*1*–*3*). Occasionally, spillover into domestic poultry or domesticated mammals can result in human infections (*2*,*4*,*5*) and sustained transmission within a new mammalian host, as shown by equine influenza virus (H3N8) (EIV) (*3*).

The H3N8 EIVs were reported in the southern United States in 1963 during an outbreak in horses imported from Argentina (*6*,*7*). This emergence resulted in a pandemic that led to international cocirculation of H7N7 and H3N8 EIVs during the 1960s and 1970s, causing heterosubtypic reassortment that might have contributed to the extinction of H7N7 EIV (*8*). Today, H3N8 EIVs represent a single genetic lineage capable of inducing serious respiratory disease in susceptible horses.

The origin of the H3N8 lineage is unknown; however, phylogenetic studies and uracil content analysis suggest that these viruses originated in wild birds (*9*). The H3N8 EIV-like polymerase acidic (PA), nucleoprotein (NP), and nonstructural (NS) genes have been identified in avian influenza viruses (AIV) isolated from South American wild birds since the mid-2000s; the most recent isolation was in Argentina during 2016 (*10**–**12*). Time to most recent common ancestor (tMRCA) analysis suggests that these genes likely originated in AIVs during the 1950s (*9*,*12*). However, an EIV-like H3 hemagglutinin (HA) has yet to be identified in AIVs from wild birds.

We performed active surveillance of wild birds in Chile and isolated 6 distinct AIVs with HA, NP, NS, and PA genes having high nucleotide homology with the 1963 H3 EIV. The AIVs were isolated from resident waterfowl belonging to the families *Anatidae* and *Rallidae*, suggesting that circulation of these viruses might be restricted to nonmigratory species found only in the Southern Cone of South America. Although viruses from Chile had nucleotide similarity with H3 EIVs, they were antigenically like avian influenza viruses and could be transmitted into chickens, suggesting adaptations to the avian host. These studies provided the initial evidence that an H3 EIV-like HA continues to circulate in wild birds.

## Materials and Methods

### Sample Collection, Screening, and Sequencing

Fresh feces from birds were collected from the environment during 2013–2017 at different wetlands across the central region of Chile. These samples were collected by using sterile flocked swabs (Copan Italia S.P.A., https://www.copangroup.com) and stored in 1-mL universal transport media tubes (Copan Italia S.P.A). They were then transported at 4°C to the Faculty of Veterinary Science of the University of Chile (Santiago, Chile) and stored at −80°C until analysis.

RNA extraction and quantitative reverse transcription PCR were performed at St. Jude Children’s Research Hospital (Memphis, TN, USA) as described (*13*). In brief, RNA was extracted from 50 μL of swab specimen by using the Mag Max-96 IA/ND Viral RNA Isolation Kit (Life Technologies, https://www.thermofisher.com) on a Kingfisher Flex Magnetic Particle Processor (ThermoFisher Scientific, https://www.thermofisher.com). Quantitative reverse transcription PCR was performed on a CFX96 Real-Time PCR System with the 4x TaqMan Fast Virus Master Mix (ThermoFisher Scientific) and primers and probes specific for the influenza A matrix gene (*14*). Samples with a cycle threshold value <38 where considered having positive results. Viral isolation was attempted on 9-day old specific pathogen–free (SPF) embryonated chicken eggs as described (*15*).

To identify the host species, genetic barcoding was performed by using primers that amplified the cytochrome oxidase I gene as described (*16*). Sequencing was performed by using either Sanger sequencing and universal oligonucleotide primer sets as described (*17*) or by deep sequencing on an Illumina MiSeq System (https://www.illumina.com) as described (*18*). Sequences were assembled by using CLC Genomic Workbench Version 9 (http://webapp.cabgrid.res.in/biocomp/CLCBio/clc-bio.html). The H3 sequences from Chile used in this study have been deposited into GenBank (accession nos. KX101146, KY644162, MH675632, MH499154, MK163999, and MK164010).

### Genetic and Phylogenetic Analysis

Phylogenetic analysis included sequences from avian and equine hosts downloaded from the National Center for Biotechnology Information (Bethesda, MD, USA) Influenza Virus Database. BLAST analysis (https://blast.ncbi.nlm.nih.gov/Blast.cgi) was performed to ensure that sequences from Chile not have higher homology to influenza A virus in other hosts. Representative sequences were selected by clustering similar sequences using CD-HIT-EST (*19*).

Maximum-likelihood trees were constructed by using the DECIPHER and PHANGORN packages in R (*20*,*21*). Sequences were aligned by using DECIPHER with the AlignTranslation function. The modelTest function was used to evaluate which nucleotide substitution with and without gamma-distributed rate variation among sites (γ) and invariant sites (I) is the best fit model. The generalized time reversible (GTR) + γ 4 + I model was best fitting by using Bayesian information criteria. The phylogenetic tree was constructed according to a standard protocol using phangorn (*21*). The starting neighbor-joining tree was constructed by using the distance matrix, and maximum-likelihood trees were generated from the starting tree by using the GTR + γ 4 + I model with stochastic branch rearrangement. The bootstrap method was used to determine the confidence of the tree topology with 1,000 replicate trees. The maximum-likelihood tree was saved with the bootstrap percentage in the Newick format (https://evolution.genetics.washington.edu) and visualized by using the ggtree package (*22*). Alternatively, the maximum-likelihood tree was constructed by using RaxML with a GTR + γ distribution with 1,000 bootstraps (*23*). The tree was visualized in FigTree version 1.4.4 (http://tree.bio.ed.ac.uk).

Clocklikeness of the resulting trees was investigated by using TempEst to determine if the molecular-clock assumption holds for the gene segment (*24*). The NS1 gene segment had no temporal signal and was not further analyzed. The H3, PA, and NP gene segments had positive, linear time vs. root-to-tip distance trend and a moderate scatter with correlation coefficients from 0.55 to 0.7. These gene segments were further analyzed to estimate the age of the most recent common ancestor.

We estimated tMRCAs by using tip date sampling and Bayesian Markov chain Monte Carlo analysis with the BEAST 1.10 package (*25*). Dates were estimated by using coalescent constant size prior with 3 partitions for the 3 codons using the maximum-likelihood estimation tree as the starting tree. Simulations were run using either the GTR or Hasegawa-Kishino-Yano substitution model and either the strict clock or the uncorrelated relaxed clock model to determine the robustness of the date estimates (*26*). The substitution and clock model had minor effects on the divergence date estimates. The maximum clade credibility trees (MCC) generated by the treeannotator package included with BEAST is reported. Figures were visualized and annotated by using the ggtree package (*22*).

### Viruses and Cells

We propagated A/California/04/2009(H1N1) and H3Nx viruses A/equine/Miami/1/1963 (H3N8), A/equine/Uruguay/1/1963(H3N8), A/equine/New York/2016(H3N8), A/equine/New York/2016(H3N8), A/red-gartered coot/Chile/C16030/2016(H3N4), A/red-fronted coot/Chile/5/2013(H3N6), A/yellow-billed pintail/Chile/C2014/2015(H3N8), A/mallard/Oregon/449221–105/2006(H3N6), A/duck/Minnesota/34/1976(H3N8), A/mallard/Wisconsin/22/1974(H3N5), A/blue-winged teal/Wisconsin/279/1975(H3N8), A/green winged teal/Alaska/292/2011(H3N8), A/northern pintail/Alaska/496/2012(H3N8), and A/northern pintail/Alaska/870/2014(H3N8) in 10-day-old embryonated chicken eggs as described (*27*). MDCK and A549 cells were cultured in modified Eagle medium (Corning 10-010-CV; https://www.corning.com) containing 200 mmol/L GlutaMAX (GIBCO 15290–026; https://www.thermofisher.com/us/en/home/brands/gibco.html) and 10% fetal bovine serum (Atlanta Biologicals, https://www.rndsystems.com). All viruses used were natural isolates.

### Hemagglutination Inhibition Assay

We tested 2 equine H3 viruses [A/equine/Uruguay/1/1963(H3N8) and A/equine/Chile/EQCL003/2018(H3N8)]; 3 wild bird viruses from Chile [A/red-gartered coot/Chile/C16030/2016(H3N4), A/red-fronted coot/Chile/5/2013(H3N6), and A/yellow-billed pintail/Chile/2015(H3N6)]; and 7 wild bird origin North American viruses [A/mallard/Oregon/449221–105/2006(H3N6), A/duck/Minnesota/34/1976(H3N8), A/mallard/Wisconsin/22/1974(H3N5), A/blue-winged teal/Wisconsin/279/1975(H3N8), A/green winged teal/Alaska/292/2011(H3N8), A/northern pintail/Alaska/496/201(H3N8), and A/northern pintail/Alaska/870/2014(H3N8)] in a hemagglutination inhibition (HI) assay against a panel of 3 ferret-generated antiserum [A/equine/Miami/1/1963(H3N8), A/equine/New York/2016(H3N8), and A/yellow-billed pintail/Chile/C2014/2015(H3N8)] according to World Health Organization guidelines (*28*). In brief, 25 μL of serum was treated overnight with receptor-destroying enzyme (Denka Seiken, Co., Ltd., https://denka-seiken.com) and serially diluted 2-fold in 25 μL of phosphate-buffered saline in duplicate in a 96 well v-bottom plate. Each homologous virus was adjusted to 4 hemagglutination units, and 25 μL were added to the serum dilutions and incubated for 30 min at 4°C. Finally, 50 μL of 0.5% chicken erythrocytes were added to each of the wells, and the plate was incubated at 4°C for 30 min, after which results of the assay were read.

### In Vitro Infections

A549 and MDCK cells were infected at a multiplicity of infection of 0.01 for 1 hour at 37°C. Cells were washed 3 times to remove unbound virus, and infected cells were cultured in appropriate medium containing 0.075% bovine serum albumin and 1 μg/mL l-1-tosylamido-2-phenylethyl chloromethyl ketone–treated trypsin. Aliquots of culture supernatants were collected at 6, 16, 24, 48, and 72 hours postinfection (hpi) and immediately stored at −80°C for the determination of virus titers by 50% median tissue culture infectious dose assay in MDCK cells as described (*29*).

### Chicken Transmission Study

The chicken experiment was performed as described (*30*). In brief, 6-week-old SPF leghorn chickens (3/group) were separated into 4 experimental groups: (A/Equine/NY/2/2016 H3N8, A/red-fronted coot/Chile/5/2013 H3N6, A/red-gartered coot/Chile/C16030/2016 H3N4, and A/red-fronted coot/Chile/5/2013 H3N6) and inoculated with 10^6 ^50% egg infective dose/0.5 mL virus by intraocular, intranasal, and intratracheal routes. These chickens were placed in direct contact with virus-naive chickens (n = 9/group) 24 hours later. All chickens were observed daily for clinical signs of illness, such as body weight loss, labored breathing (including upper respiratory signs, such as coughing and sneezing), and diarrhea. To assess virus shedding, cloacal and tracheal swab specimens were collected at 3, 5, 7, 9, and 12 dpi. Swab specimens were stored in viral transport medium at −80°C until virus titration. Viral titers were established by using the method of Reed and Munch by performing 50% median tissue culture infectious dose assay analysis of the swab specimen inoculum with 50 μL of allantoic fluid and 50 μL of 0.5% chicken erythrocytes (*29*). All birds were euthanized at day 21 pi, and 1 mL blood was collected of each bird to check for seroconversion by HI. Chicken serum samples were tested with their corresponding homologous virus, according to their assigned group.

### Statistical Analysis and Ethics

Mean infectious titers were analyzed by 2-way analysis of variance using GraphPad Prism version 8 ( https://www.graphpad.com). Area under the curve analysis for measuring cumulative shedding was performed by using GraphPad Prism version 8. All animal experiments, procedures, and sampling activities were approved by the St. Jude Children’s Research Hospital Institutional Animal Care and Use Committee.

## Results

### HA Gene Phylogenetic Nucleotide Analysis

**Figure 1:**
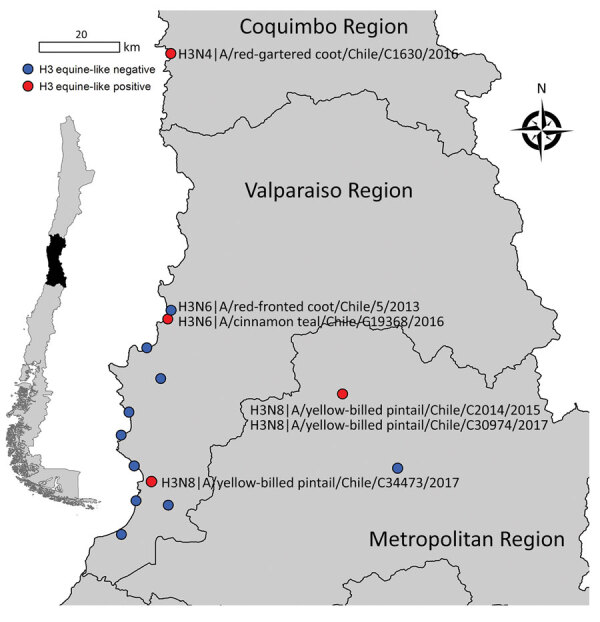
Central region of Chile showing where different equine-like H3Nx influenza viruses were obtained (red dots). Blue dots indicate other avian influenza virus surveillance sites. Isolate names and subtypes are indicated. Inset map indicates location of study area within Chile.

**Figure 2:**
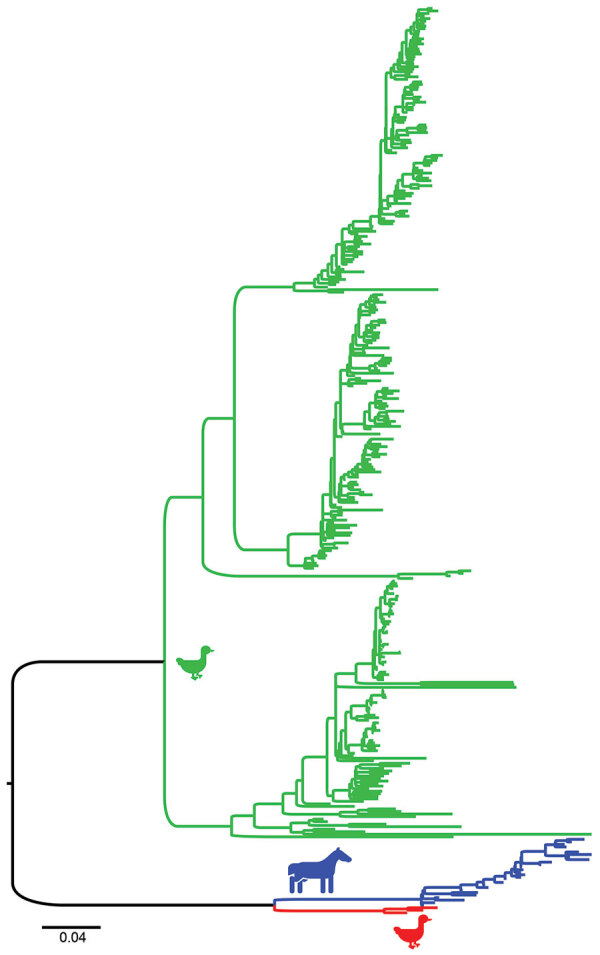
Maximum-likelihood phylogenetic tree showing the relationship between equine influenza (H3N8) viruses (blue), equine-like avian influenza viruses (AIVs) from Chile (red), and AIVs from other locations (green) for the H3 gene fragment. Scale bars indicate average nucleotide substitutions per site. A complete tree, taxon identification, and bootstrap support are shown in Appendix Figure 1.

**Figure 3:**
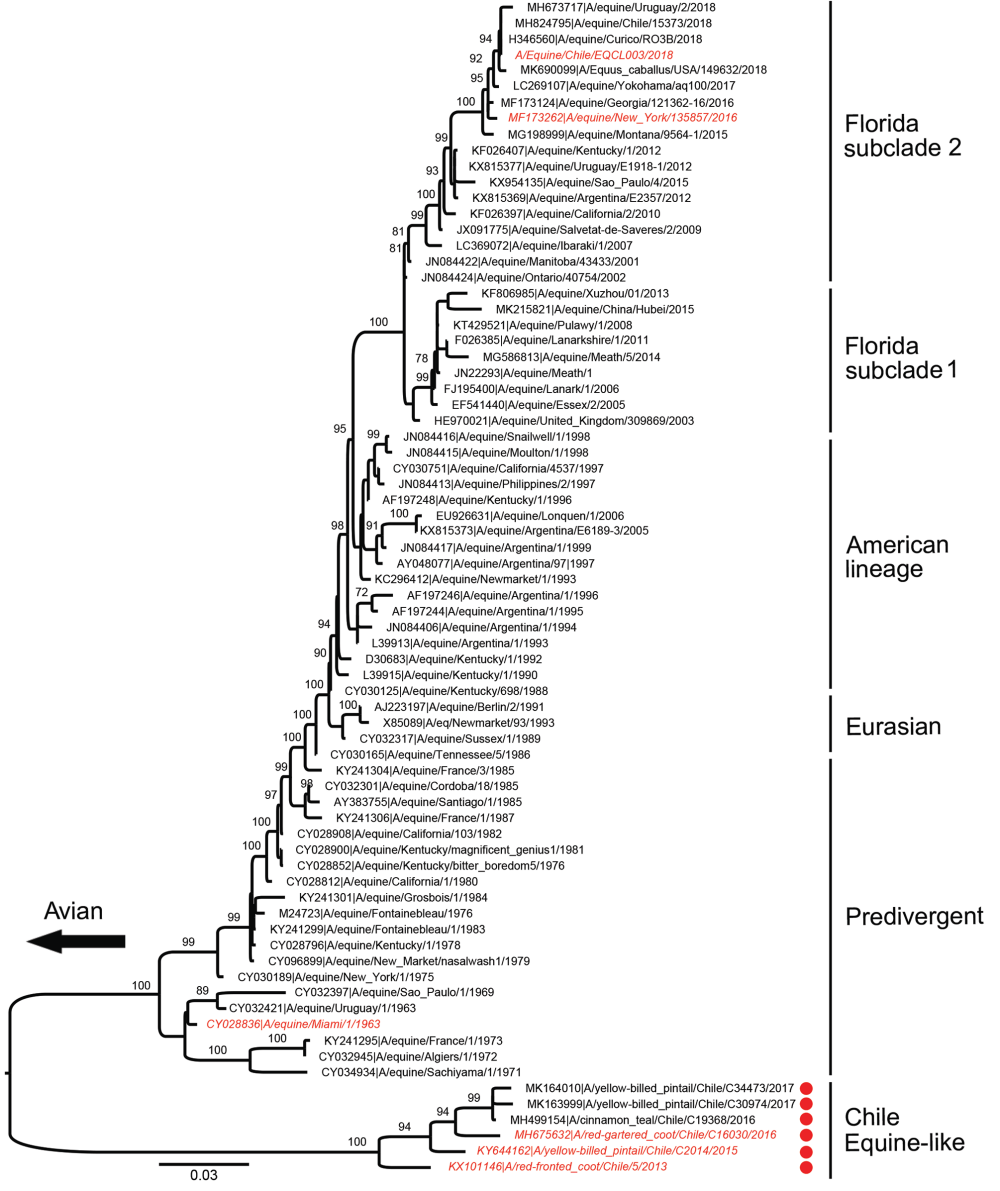
Maximum-likelihood phylogenetic analysis of the hemagglutinin gene of equine-like influenza (H3N8) viruses from Chile sequenced for this study (red dots) and reference sequences. Bootstrap values >70 are indicated. Viruses used in antigenic studies are indicated in red and italics. Major equine-like avian influenza virus clades are shown. Scale bars indicate average nucleotide substitutions per site.

During 2013–2017, a total of 37,171‬ wild bird fresh fecal samples were collected from various wetlands across Chile. H3 subtypes accounted for 5.8% (n = 8) of the total HA diversity. Six of the H3 viruses, A/cinnamon teal/Chile/C19368/2016 (H3N8), A/red-fronted coot/Chile/5/2013 (H3N6), A/yellow-billed pintail/Chile/C2014/2015 (H3N8), A/red-gartered coot/Chile/C16030/2016 (H3N4), A/yellow-billed pintail/Chile/C30974/2017 (H3N8), and A/yellow-billed pintail/Chile/C34473/2017 (H3N8), were isolated from different regions of Chile (Figure 1), had highest nucleotide homology with the HA gene of the 1963 H3N8 EIV lineage, and had lower homology to the H3 of AIVs found in other regions (Figure 2; Appendix Figure 1). Maximum-likelihood trees demonstrated that the H3 HA sequences form 4 distinct clusters, with 3 clusters for wild bird origin viruses and 1 cluster for 1963 H3 EIV. The 6 IAVs for Chile form a sister clade relationship with the 1963 H3 EIV and are more phylogenetically related to the 1963 H3N8 EIV than to other avian H3 HAs (Figure 3; Appendix Figure 1).

**Figure 4:**
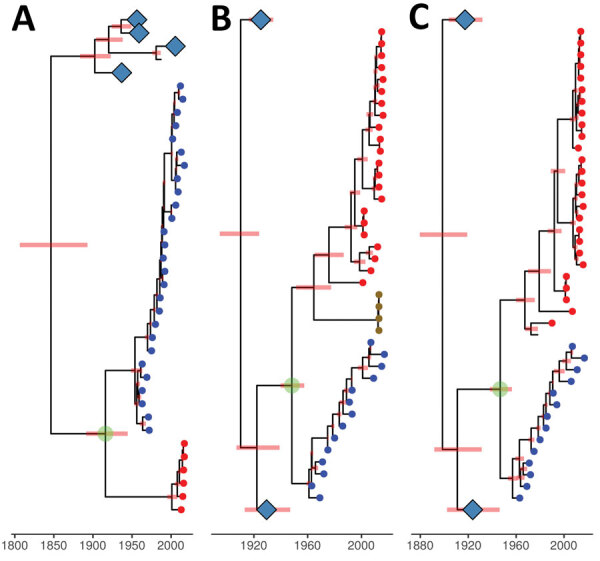
Genetic relationship and divergence date between avian influenza viruses (AIVs) from Chile/South America, the 1963 H3N8 equine-like influenza virus (EIV), and AIVs from other locations. A) Maximum clade credibility tree for the H3 gene segment. B) Maximum clade credibility tree for the polymerase acidic gene segment. AIV samples for penguins from Antarctica are represented by gold circular nodes. C) Maximum clade credibility tree for the nucleoprotein gene segment. Viruses were dated by using Bayesian Markov chain Monte Carlo analysis. Position of tips represent sampled virus for the years they were sampled. H3N8 EIV sequences are represented by blue circular nodes, AIVs from Chile/South America are represented by red circular nodes, and avian sequences from other locations are unlabeled. Internal nodes are reconstructed common ancestors, and pink bars represent 95% credible intervals on their date. Large clades of avian sequences from other locations are collapsed on their common ancestors and represented by light blue diamonds. The common ancestor between most AIVs from South America and the 1963 H3N8 EIV is highlighted by a green circle. Time scale bar indicates years. .

Bayesian molecular clock analysis was used to estimate the tMRCA between the H3 AIVs from Chile and 1963 H3 EIV, and between avian H3 sequences not from Chile and EIV H3 (Table 1, Figure 4, panel A). The tMRCA of the 1963 EIV H3 is 1954 (95% credible interval 1948–1959). The tMRCA of the H3 HA not from Chile and the 1963 EIV H3 HA is 1916 (95% credible interval 1883–1941). In contrast, the tMRCA of avian H3 HAs not from Chile and the H3/1963 H3N8 EIV from Chile is 1845 (95% credible interval 1795–1882). We provide a complete HA divergence time tree (Appendix Figure 2).

**Table 1:** Divergence dates per gene segment for equine-like H3 avian influenza viruses in wild birds, Chile*

Gene segment	Equine-like, no. positive/no. tested†	GTR relaxed	GTR strict	HKY relaxed	HKY strict
HA	6/8	1916 (1883–1941)	1918 (1909–1927)	1917 (1886–1939)	1918 (1909–1927)
PA	19/26	1948 (1938–1956)	1946 (1941–1950)	1948 (1935–1957)	1946 (1941–1950)
NP	24/29	1947 (1936–1955)	1945 (1940–1950)	1947 (1936–1955)	1945 (1939–1949)
NS1	11/27	NA	NA	NA	NA

*Estimated divergence dates between these sequences from Chile and the 1963 H3N8 equine influenza sequences were estimated by using BEAST version 1.10 (https://beast.community/2018-06-10_BEAST_v1.10.0_released.html) with GTR or HKY substitution models and strict or uncorrelated relaxed clocks. Estimated dates are reported with 95% credible interval dates in parentheses. Because NS1 sequences did not exhibit molecular clock–like behavior, the divergence date could not be estimated. GTR, generalized time reversible; HA, hemagglutinin; HKY, Hasegawa-Kishino-Yano; NA, not available; NP, nucleoprotein; NS1, nonstructural protein 1; PA, polymerase acidic. †Number of sequences from Chile for the gene segments that have high homology to the 1963 H3N8 equine influenza virus.

### Internal Gene Phylogenetic Nucleotide Analysis

The nucleotide similarity with the EIV H3 HA prompted us to conduct a detailed phylogenetic analysis. Like HA, the H3 viruses from Chile had NS1 (Appendix Figure 3), NP (Appendix Figure 4), and PA (Appendix Figure 5) genes that were phylogenetically more like EIVs than avian viruses at the nucleotide level. Most of the NS1, NP, and PA gene segments obtained from IAVs from South America formed a sister clade relationship with 1963 H3N8 EIV (Appendix Figures 3–5). There was 1 NP sequence collected from Canada (A/blue-winged teal/ALB/651/1978 (H6N2), GenBank accession no. ABB18989) that was more like these sequences from South America than other AIVs, suggesting that these gene fragments have spread into North America through bird migration, but did not establish a major reservoir to be consistently identified during surveillance. The NA, matrix, polymerase basic 1, and polymerase basic 2 gene segments showed greater similarities to typically avian origin gene segments (Appendix Figures 6, 7) and were not further analyzed.

Bayesian molecular clock analysis was performed for the NP and PA gene segments to estimate the age of tMRCA between the 1963 H3 EIV and sequences from Chile (Table 1; Figure 4, panels B, C). The tMRCA of the NP gene segment between most of the sequences from Chile and the 1963 H3N8 EIV is 1947 (95% credible interval 1936–1955) (Appendix Figure 8). The tMRCA for the PA gene is 1948 (95% credible interval 1938–1956) (Appendix Figure 9).

The NS1 segment had insufficient temporal signal for molecular clock analysis. Overall, our phylogenetic analysis suggests that many of the gene segments collected from wild birds in South America were distinct from those collected from wild birds in North America; the H3, PA, NP, and NS1 genes were more closely related to the 1963 H3N8 EIV, supporting the hypothesis that the 1963 H3N8 EIV originated from wild birds in South America. Our results are consistent with those of Rimondi et al., who reported that viruses from birds from Argentina also carry PA and NP sequences with homology to the EIV (*12*). These sequences are closely related to the highlighted sequences from Chile. Rimondi et al. also estimated the tMRCA for PA to be 1943, and the tMRCA for NP to be 1951, which were similar to our estimates.

### Antigenicity and Biologic Properties

The H3 HA from Chile and the 1963 EIV H3 HA also show amino acid similarity, having 91.5% amino acid identity to A/equine/Uruguay/1/1963. Thus, antigenicity was assessed by using the HI assay with ferret antisera against equine viruses or viruses from Chile that was available for these studies. The H3 viruses from Chile were not inhibited by ferret-generated antisera against A/equine/Miami/1963 or A/equine/New York/2016 viruses (Table 2). The equine viruses also failed to cross-react with ferret antisera generated against A/yellow-billed pintail/Chile/C2014/2015 virus (Table 2). Antisera against the avian virus from Chile or equine virus did not inhibit H3 IAVs representing the 3 genetic wild bird clusters identified (Appendix Figure 1). These studies highlight that the H3 viruses from Chile are antigenically unique from equine and other wild bird origin H3 IAVs.

**Table 2:** Hemagglutination inhibition assay used for analysis of equine-like H3 avian influenza viruses in wild birds, Chile*

Virus	Subtype	Ferret antiserum titers		
		eq/Miami/1963	eq/NY/2016	YBP/Chile/2015
A/equine/Miami/1/1963	H3N8	**1:640**	<1:10	<1:10
A/equine/NY/2016	H3N8	1:1,280	**1:640**	<1:10
A/yellow-billed pintail/Chile/2015	H3N6	<1:10	<1:10	**1:160**
Test viruses				
A/equine/Uruguay/1/1963	H3N8	1:320	<1:10	<1:10
A/equine/Chile/EQCL003/2018	H3N8	1:640	1:320	NT
A/red-fronted coot/Chile/5/2013	H3N6	<1:10	<1:10	1:160
A/red-gartered coot/Chile/C16030/2016	H3N4	<1:10	<1:10	1:80
A/mallard/Oregon/449221–105/2006	H3N6	<1:10	<1:10	<1:10
A/duck/Minnesota/34/1976	H3N8	<1:10	<1:10	<1:10
A/mallard/Wisconsin/22/1974	H3N5	<1:10	<1:10	<1:10
A/blue winged teal/Wisconsin/279/1975	H3N8	<1:10	<1:10	<1:10
A/green winged teal/Alaska/292/2011	H3N8	<1:10	<1:10	<1:10
A/northern pintail/Alaska/496/2012	H3N8	<1:10	<1:10	<1:10
A/northern pintail/Alaska/870/2014	H3N8	<1:10	<1:10	<1:10

*Bold indicates homologous serum inhibition values. eq/Miami/1963, A/equine/Miami/1/1963; eq/NY/2016, A/equine/NY/2016; YBP/Chile/2015, A/yellow-billed pintail/Chile/2015; NT, not tested.

**Figure 5:**
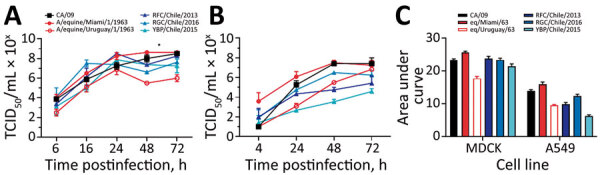
Replicative capacity of H3Nx influenza viruses in vitro. A, B) To evaluate the replication of H3N8 viruses in vitro, MDCK cells (A) and human lung A549 cells (B) were infected at a multiplicity of infection of 0.01, and cell culture supernatants were collected at 6, 16, 24, 48, and 72 hours postinfection. Viral titers were determined by TCID_50_ analysis in triplicate. Values are mean titers of 3 replicates, and error bars indicate SEMs. Differences were considered significant at p<0.05 (*). C) Cumulative shedding for each cell line and each viral strain shown. TCID_50_, 50% median tissue culture infectious dose.

To compare biologic properties, we assessed virus growth in mammalian cells and infections in vivo. The viruses from Chile and 1963 EIV H3 viruses replicated to similar titers and kinetics in MDCK cells (except at 48 hpi) when the eq/Miami/63 virus replicated to significantly higher titers compared with the other H3 viruses (p<0.05) (Figure 5, panel A). There was no major difference between eq/Miami/63 virus and the control A/California/2009 H1N1 virus. Replication kinetics and titers were significantly different in human A549 cells in which eq/Miami/63 virus again replicated to significantly higher titers at 24 hpi and 48 hpi (p<0.05) (Figure 5, panel B). The IAVs from Chile had lower overall titers and slower kinetics compared with those of eq/Miami/63 virus. This finding is highlighted by area under the curve analysis (Figure 5, panel C). In contrast to the results with eq/Miami/63 virus, the IAVs from Chile had higher viral titers from 24 hpi to 72 hpi than eq/Uruguay/63 virus, which is genetically similar to eq/Miami/63 virus (p<0.05) (Figure 5, panel A). A/red-footed Coot/Chile/5/2013 virus also replicated to higher titers than the eq/Uruguay/63 virus in A549 cells (p<0.05) (Figure 5, panel B). The reasons for the differential replication kinetics between the genetically similar equine viruses is unknown but was consistent. Attempts to replicate the viruses in EQKC3 and NBL6 equine cell lines were not successful.

In vivo, the equine-like H3 viruses readily infected and transmitted in experimentally infected chicken. In brief, 4 groups of 6 week of age SPF chickens were inoculated with 10^6 ^50% egg infective dose/0.5 mL virus (n = 3 per group) and then placed in direct contact with virus-naive chickens (n = 9 per group) 24 hours later. Oropharyngeal and cloacal viral shedding was monitored for 12 days postinfection (dpi), and seroconversion was measured at 21 dpi. At 3 dpi, each group infected with H3 virus from Chile had at >1 donor bird showing oropharyngeal virus shedding. The group inoculated with A/red-footed Coot/Chile/5/2013(H3N6) (RF Coot) virus also showed shedding by the cloacal route at this time point. After 5 dpi, donors were shedding virus by mixed oropharyngeal and cloacal routes. After 7 dpi, only shedding by the cloacal route was detected in the group inoculated with A/red-gartered coot/Chile/C16030/2016 virus (RG Coot H3N4) (Table 3). Although initially all 3 groups showed similar infection and transmission (3/3 infected donors and 3–5 infected direct contacts), the direct contact group infected with A/red-fronted coot/Chile/5/2013 virus (RF Coot H3N6) was the only group showing persistent cloacal shedding of virus in 1 of the direct contacts until the end of the experiment at 12 dpi. Equally, RG Coot H3N4 showed cloacal shedding of virus in 1 of the donors until 12 dpi.

**Table 3:** Infection and transmission in chickens of equine-like H3 avian influenza viruses in wild birds, Chile*

Bird, virus subtype	Type of infection	3 dpi			5 dpi			7 dpi			9 dpi			12 dpi		HI titer, 21 dpi
		OP	CL		OP	CL		OP	CL		OP	CL		OP	CL	
PBS, n = 6	C	0/6	0/6		0/6	0/6		0/6	0/6		0/6	0/6		0/6	0/6	0/6
eq/Miami/1963, H3N8	I	0/3	0/3		0/3	0/3		0/3	0/3		0/3	0/3		0/3	0/3	0/3
	DC	0/9	0/9		0/9	0/9		0/9	0/9		0/9	0/9		0/9	0/9	0/9
eq/NY/2016, H3N8	I	0/3	0/3		0/3	0/3		0/3	0/3		0/3	0/3		0/3	0/3	1/3 (160)
	DC	0/9	0/9		0/9	0/9		0/9	0/9		0/9	0/9		0/9	0/9	0/9
Red-fronted coot, H3N6	I	3/3 (2.75– 5.5)	1/3 (8.25)		2/3 (2.25–4.25)	0/3		0/3	0/3		0/3	0/3		0/3	0/3	2/3 (20–80)
	DC	4/9 (3.25–4.5)	0/9		5/9 (2.75–5.75)	1/9 (7.75)		3/9 (2.5–5.5)	1/9 (8.5)		2/9 (2.25–4.75)	1/9 (2.75)		0/9	1/9 (2)	2/9 (20)
Yellow-billed pintail, H3N8	I	3/3 (2.5–5.5)	0/3		1/3 (3.25)	0/3		0/3	0/3		0/3	0/3		0/3	0/3	2/3 (40–80)
	DC	3/9 (3.5–5.5)	1/9 (5.5)		3/9 (2.75–5.5)	1/9 (6.5)		1/9 (5)	1/9 (5.25)		0/9	1/9 (2.75)		0/9	0/9	1/9 (40)
Red-gartered coot, H3N4	I	3/3 (2.25)	0/3		1/3 (2.25)	1/3 (5.5)		0/3	1/3 (5.5)		0/3	1/3 (3.75)		0/3	1/3 (3.25)	2/3 (20–40)
	DC	5/9 (2.25–4)	1/9 (2.25)		4/9 (3–5.5)	0/9		2/9 (3.75–4)	0/9		0/9	0/9		0/9	0/9	4/9 (20–40)

*Values are no. birds infected/total number in group. Values in parentheses are ranges of viral titers (log 50% median tissue culture infectious dose) per timepoint and group. C, control; CL, cloacal; DC, direct contact; dpi, days postinfection; H, hemagglutinin; HI, hemagglutinin inhibition; I, inoculates; N, neuraminidase; OP, oropharyngeal; PBS, phosphate-buffered saline.

Seroconversion, as measured by HI titers at 21 dpi, was similar across all experimental groups and titers ranged from 1:20 to 1:80. In contrast, the eq/Miami/63 or the equine/NY/2016 viruses did not infect or were transmitted in chickens. However, 1 eq/NY/2016 donor chicken did have HI titers (1:60) at 21 dpi, suggesting infection. Overall, these studies demonstrate that there are H3 IAVs circulating in wild birds in Chile that are antigenically unique from H3 EIV and other wild bird AIVs and are capable of infecting and transmitting in poultry.

## Discussion

In these studies, we isolated several H3 influenza viruses from wild birds in Chile that contain HA, NP, PA, and NS segments that are genetically similar to the 1963 EIVs, suggesting that the direct descendants of the virus that originated the EIV pandemic continue to circulate in wild birds in Chile. Previous AIV surveillance studies in South America have identified internal genes that were putatively related to the ancestral virus that originated the 1963 equine pandemic (*9*,*13*,*31*); however, we have identified an EIV-like HA in wild birds. Although phylogenetic nucleotide sequence analysis places the H3 viruses from Chile in a monophyletic group together with equine viruses, amino acid composition of the HA and antigenic properties show that viruses from Chile are antigenically unique and can infect and transmit in poultry. However, to better interpret the antigenic data, future studies could benefit from the use of antigenic cartography to learn about the antigenic evolution of this subtype (*32*).

Although H3 is the most common subtype found in wild birds from North America (*33*), typical avian origin H3 subtypes are seldom recovered in South America. To date, only 4 isolates in Peru and 2 in Chile, all resembling contemporary North American AIVs, have been described (*31*,*34*). This finding could be related to the lack of surveillance throughout Latin America or low-level circulation of H3 viruses in wild birds (*35*). In contrast, we found that the H13 and H16 subtypes comprised up to 54% of the overall subtype diversity of AIVs from Chile deposited in GenBank during the same surveillance period (2013–2017), which could be caused by a biased surveillance effort in the order Charadriiformes. However, if we only consider isolates obtained from waterfowl in South America, the abundance of the H3 subtype (8.5%) is below the relative abundance of more common AIV subtypes, such as H1 (10.6%), H4 (10.6%), H5 (17%), and H7 (14.9%), which illustrates rarity of H3 subtype virus in wild birds in South America.

Global trade of thoroughbred horses from South America carrying the original 1963 H3N8 EIV, and not migratory birds, was responsible for the spread of these avian-origin gene segments (*36*). In comparison, although independent avian-to-equine transmission of lineage H3N8 AIV from Asia to horses was described in eastern Asia in the late 1980s, the epizootic event that followed was self-limited and died out after a few years (*37*). Subsequent active surveillance of wild birds in Mongolia has shown that AIVs carrying several gene segments closely related to H3N8 EIV from Asia are still circulating in wild birds, similar to our findings in Chile (*38*). This finding suggests that avian-to-equine transmission of H3 influenza A viruses is not an uncommon event, but that posterior sustained transmission is more limited. The conditions and key genetic signatures that facilitated the species-jump and rapid adaptation from waterfowl to horses in South America and eastern Asia remain unknown. This limitation reflects our inability to estimate whether the EIV-like HA reassorted together with the PA/NP/NS1 or in a separate event on the basis of our data. However, it is not uncommon to find wild waterfowl next to free-ranging horses in South America, which enables repeated transmission events that might lead to emergence of a new virus strain with pandemic potential in horses.

In summary, our data provide evidence that gene segments, including HA, that are the closest ancestor of the 1963 H3N8 EIV continue to circulate in wild bird reservoirs. We recommend increased surveillance to better clarify the role of this subtype in the context of genetic diversity of IAVs in South America, its epidemiology and ecology, and the risk that this new subtype represents to avian and mammalian hosts.

Appendix Additional information on equine-like H3 avian influenza viruses in wild birds, Chile.

## References

[1] Webster RG, Bean WJ, Gorman OT, Chambers TM, Kawaoka Y. Evolution and ecology of influenza A viruses. Microbiol Rev. 1992;56:152–79. 10.1128/MMBR.56.1.152-179.1992 1579108 PMC372859

[2] Webster RG, Govorkova EA. Continuing challenges in influenza. Ann N Y Acad Sci. 2014;1323:115–39. 10.1111/nyas.12462 24891213 PMC4159436

[3] Parrish CR, Murcia PR, Holmes EC. Influenza virus reservoirs and intermediate hosts: dogs, horses, and new possibilities for influenza virus exposure of humans. J Virol. 2015;89:2990–4. 10.1128/JVI.03146-14 25540375 PMC4337525

[4] Joseph U, Su YC, Vijaykrishna D, Smith GJ. The ecology and adaptive evolution of influenza A interspecies transmission. Influenza Other Respir Viruses. 2017;11:74–84. 10.1111/irv.12412 27426214 PMC5155642

[5] Kahn RE, Ma W, Richt JA. Swine and influenza: a challenge to one health research. Curr Top Microbiol Immunol. 2014;385:205–18. 10.1007/82_2014_392 25005926

[6] Scholtens RG, Steele JH, Dowdle WR, Yarbrough WB, Robinson RQ. Epizootic of equine influenza, 1963. Public Health Rep. 1964;79:393–402. 10.2307/4592142 14153655 PMC1915427

[7] Waddell GH, Teigland MB, Sigel MM. A new influenza virus associated with equine respiratory disease. J Am Vet Med Assoc. 1963;143:587–90. 14077956

[8] Murcia PR, Wood JL, Holmes EC. Genome-scale evolution and phylodynamics of equine H3N8 influenza A virus. J Virol. 2011;85:5312–22. 10.1128/JVI.02619-10 21430049 PMC3094979

[9] Worobey M, Han GZ, Rambaut A. A synchronized global sweep of the internal genes of modern avian influenza virus. Nature. 2014;508:254–7. 10.1038/nature13016 24531761 PMC4098125

[10] Pereda AJ, Uhart M, Perez AA, Zaccagnini ME, La Sala L, Decarre J, et al. Avian influenza virus isolated in wild waterfowl in Argentina: evidence of a potentially unique phylogenetic lineage in South America. Virology. 2008;378:363–70. 10.1016/j.virol.2008.06.010 18632129 PMC2570041

[11] Spackman E, McCracken KG, Winker K, Swayne DE. An avian influenza virus from waterfowl in South America contains genes from North American avian and equine lineages. Avian Dis. 2007;51(Suppl):273–4. 10.1637/7529-032106R.1 17494565

[12] Rimondi A, Gonzalez-Reiche AS, Olivera VS, Decarre J, Castresana GJ, Romano M, et al. Evidence of a fixed internal gene constellation in influenza A viruses isolated from wild birds in Argentina (2006-2016). Emerg Microbes Infect. 2018;7:194. 10.1038/s41426-018-0190-2 30482896 PMC6258671

[13] Karlsson EA, Ciuoderis K, Freiden PJ, Seufzer B, Jones JC, Johnson J, et al. Prevalence and characterization of influenza viruses in diverse species in Los Llanos, Colombia. Emerg Microbes Infect. 2013;2:e20. 10.1038/emi.2013.20 26038461 PMC3636595

[14] Hoffmann E, Stech J, Guan Y, Webster RG, Perez DR. Universal primer set for the full-length amplification of all influenza A viruses. Arch Virol. 2001;146:2275–89. 10.1007/s007050170002 11811679

[15] Moresco KA, Stallknecht DE, Swayne DE. Evaluation and attempted optimization of avian embryos and cell culture methods for efficient isolation and propagation of low pathogenicity avian influenza viruses. Avian Dis. 2010;54(Suppl):622–6. 10.1637/8837-040309-Reg.1 20521704

[16] Cheung PP, Leung YH, Chow C-K, Ng C-F, Tsang C-L, Wu Y-O, et al. Identifying the species-origin of faecal droppings used for avian influenza virus surveillance in wild-birds. J Clin Virol. 2009;46:90–3. 10.1016/j.jcv.2009.06.016 19604718 PMC2765912

[17] Jiménez-Bluhm P, Karlsson EA, Ciuoderis KA, Cortez V, Marvin SA, Hamilton-West C, et al. Avian H11 influenza virus isolated from domestic poultry in a Colombian live animal market. Emerg Microbes Infect. 2016;5:e121. 10.1038/emi.2016.121 27924808 PMC5180366

[18] Kaplan BS, Russier M, Jeevan T, Marathe B, Govorkova EA, Russell CJ, et al. Novel highly pathogenic avian A (H5N2) and A (H5N8) influenza viruses of clade 2.3. 4.4 from North America have limited capacity for replication and transmission in mammals. MSphere. 2016;1:e00003–00016. 10.1128/mSphere.00003-16 27303732 PMC4894690

[19] Li W, Godzik A. Cd-hit: a fast program for clustering and comparing large sets of protein or nucleotide sequences. Bioinformatics. 2006;22:1658–9. 10.1093/bioinformatics/btl158 16731699

[20] Wright ES. DECIPHER: harnessing local sequence context to improve protein multiple sequence alignment. BMC Bioinformatics. 2015;16:322. 10.1186/s12859-015-0749-z 26445311 PMC4595117

[21] Schliep KP. phangorn: phylogenetic analysis in R. Bioinformatics. 2011;27:592–3. 10.1093/bioinformatics/btq706 21169378 PMC3035803

[22] Yu G, Smith DK, Zhu H, Guan Y, Lam TTY. ggtree: an R package for visualization and annotation of phylogenetic trees with their covariates and other associated data. Methods Ecol Evol. 2017;8:28–36. 10.1111/2041-210X.12628

[23] Stamatakis A. RAxML version 8: a tool for phylogenetic analysis and post-analysis of large phylogenies. Bioinformatics. 2014;30:1312–3. 10.1093/bioinformatics/btu033 24451623 PMC3998144

[24] Rambaut A, Lam TT, Max Carvalho L, Pybus OG. Exploring the temporal structure of heterochronous sequences using TempEst (formerly Path-O-Gen). Virus Evol. 2016;2:vew007. 10.1093/ve/vew007 27774300 PMC4989882

[25] Drummond AJ, Rambaut A. BEAST: Bayesian evolutionary analysis by sampling trees. BMC Evol Biol. 2007;7:214. 10.1186/1471-2148-7-214 17996036 PMC2247476

[26] Hasegawa M, Kishino H, Yano T. Dating of the human-ape splitting by a molecular clock of mitochondrial DNA. J Mol Evol. 1985;22:160–74. 10.1007/BF02101694 3934395

[27] Cline TD, Karlsson EA, Seufzer BJ, Schultz-Cherry S. The hemagglutinin protein of highly pathogenic H5N1 influenza viruses overcomes an early block in the replication cycle to promote productive replication in macrophages. J Virol. 2013;87:1411–9. 10.1128/JVI.02682-12 23152519 PMC3554171

[28] World Health Organization. Manual for the laboratory diagnosis and virological surveillance of influenza. Geneva: The Organization; 2011.

[29] Reed LJ, Muench H. A simple method of estimating fifty per cent endpoints. Am J Epidemiol. 1938;27:493–7. 10.1093/oxfordjournals.aje.a118408

[30] Jimenez-Bluhm P, Bravo-Vasquez N, Torchetti MK, Killian ML, Livingston B, Herrera J, et al. Low pathogenic avian influenza (H7N6) virus causing an outbreak in commercial Turkey farms in Chile. Emerg Microbes Infect. 2019;8:479–85. 10.1080/22221751.2019.1595162 30924394 PMC6456847

[31] Jiménez-Bluhm P, Karlsson EA, Freiden P, Sharp B, Di Pillo F, Osorio JE, et al. Wild birds in Chile Harbor diverse avian influenza A viruses. Emerg Microbes Infect. 2018;7:44. 10.1038/s41426-018-0046-9 29593259 PMC5874252

[32] Smith DJ, Lapedes AS, de Jong JC, Bestebroer TM, Rimmelzwaan GF, Osterhaus AD, et al. Mapping the antigenic and genetic evolution of influenza virus. Science. 2004;305:371–6. 10.1126/science.1097211 15218094

[33] Bahl J, Krauss S, Kühnert D, Fourment M, Raven G, Pryor SP, et al. Influenza a virus migration and persistence in North American wild birds. PLoS Pathog. 2013;9:e1003570. 10.1371/journal.ppat.1003570 24009503 PMC3757048

[34] Nelson MI, Pollett S, Ghersi B, Silva M, Simons MP, Icochea E, et al. The genetic diversity of influenza A viruses in wild birds in Peru. PLoS One. 2016;11:e0146059. 10.1371/journal.pone.0146059 26784331 PMC4718589

[35] Neumann G, Kawaoka Y. Predicting the next influenza pandemics. J Infect Dis. 2019;219(Suppl_1):S14–20. 10.1093/infdis/jiz040 30715371 PMC6452319

[36] Scholtens RG, Steele JH, Dowdle WR, Yarbrough WB, Robinson RQ. US epizootic of equine influenza, 1963. Public Health Rep. 1964;79:393–402. 10.2307/4592142 14153655 PMC1915427

[37] Guo Y, Wang M, Kawaoka Y, Gorman O, Ito T, Saito T, et al. Characterization of a new avian-like influenza A virus from horses in China. Virology. 1992;188:245–55. 10.1016/0042-6822(92)90754-D 1314452

[38] Zhu H, Damdinjav B, Gonzalez G, Patrono LV, Ramirez-Mendoza H, Amat JAR, et al. Absence of adaptive evolution is the main barrier against influenza emergence in horses in Asia despite frequent virus interspecies transmission from wild birds. PLoS Pathog. 2019;15:e1007531. 10.1371/journal.ppat.1007531 30731004 PMC6366691

