# Supplementary material for: Equine-Like H3 Avian Influenza Viruses in Wild Birds, Chile
Source: Emerg Infect Dis. 2020 Dec;26(12):2887–98. doi: 10.3201/eid2612.202063 (PMC7706983; doi:10.3201/eid2612.202063)
Supplement: Appendix — Additional information on equine-like H3 avian influenza viruses in wild birds, Chile. [file 20-2063-Techapp-s1.pdf]

Article DOI: https://doi.org/10.3201/eid2612.202063

Equine-Like H3 Avian Influenza Viruses in Wild Birds, Chile

Appendix

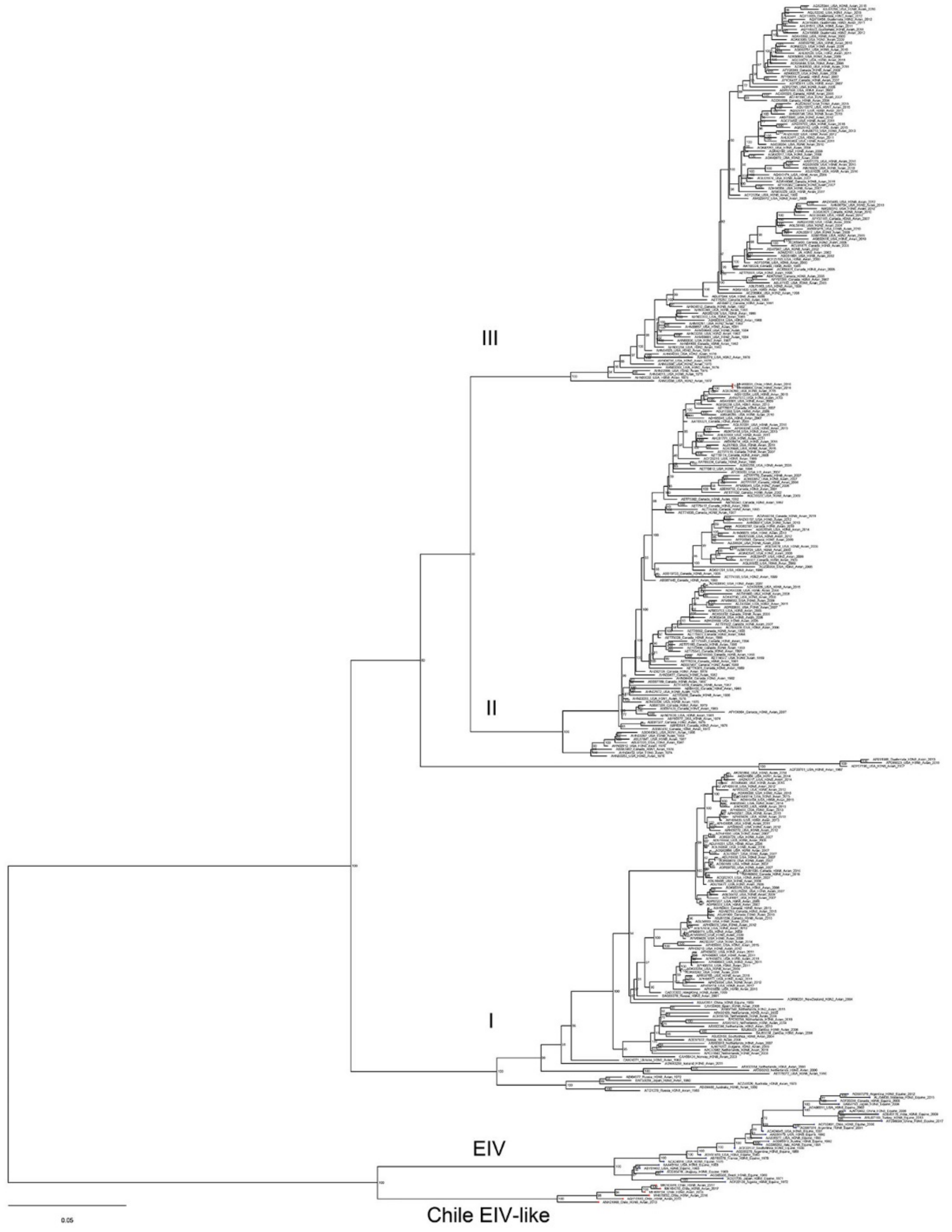

Appendix Figure 1. Maximum-likelihood phylogenetic tree showing the relationship between the H3N8 equine-like influenza virus (EIV) (blue node), avian influenza virus (AIV) from Chile (red node), and AIV samples from other locations for the gene fragment hemagglutinin (HA). Bootstrap values are labeled next to nodes. Samples from Chile and South America have greater homology to H3N8 EIV in the H3, nucleoprotein, polymerase acidic,

and nonstructural protein 1 gene segments. Major wild-bird origin H3 clusters are indicated (I, II, III), as well as EIV and EIV-like lineages from Chile. Scale bar indicates average nucleotide substitutions per site.

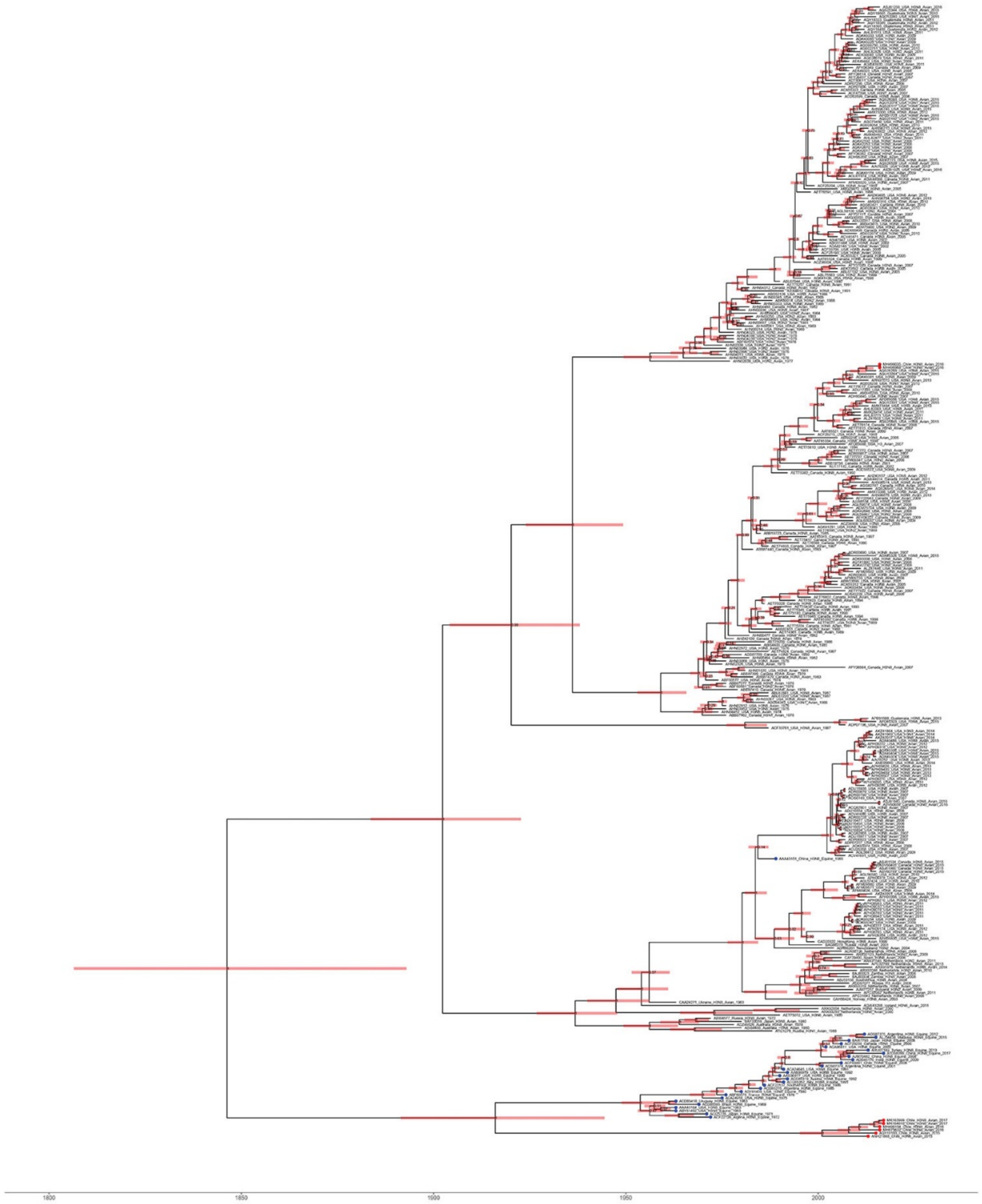

Appendix Figure 2. Maximum clade credibility tree showing the relationship between the H3N8 equine-like influenza virus (EIV) (blue node), avian influenza virus (AIV) from Chile (red node), and AIV samples from other locations for the gene fragment hemagglutinin (HA). The x-position of the tips represent the year that the virus was sampled. The x-position of the nodes corresponds to the divergence date, with the 95% highest posterior density values shaded in light red. The posterior probability values are labeled next to the nodes. Time scale bar indicates years.

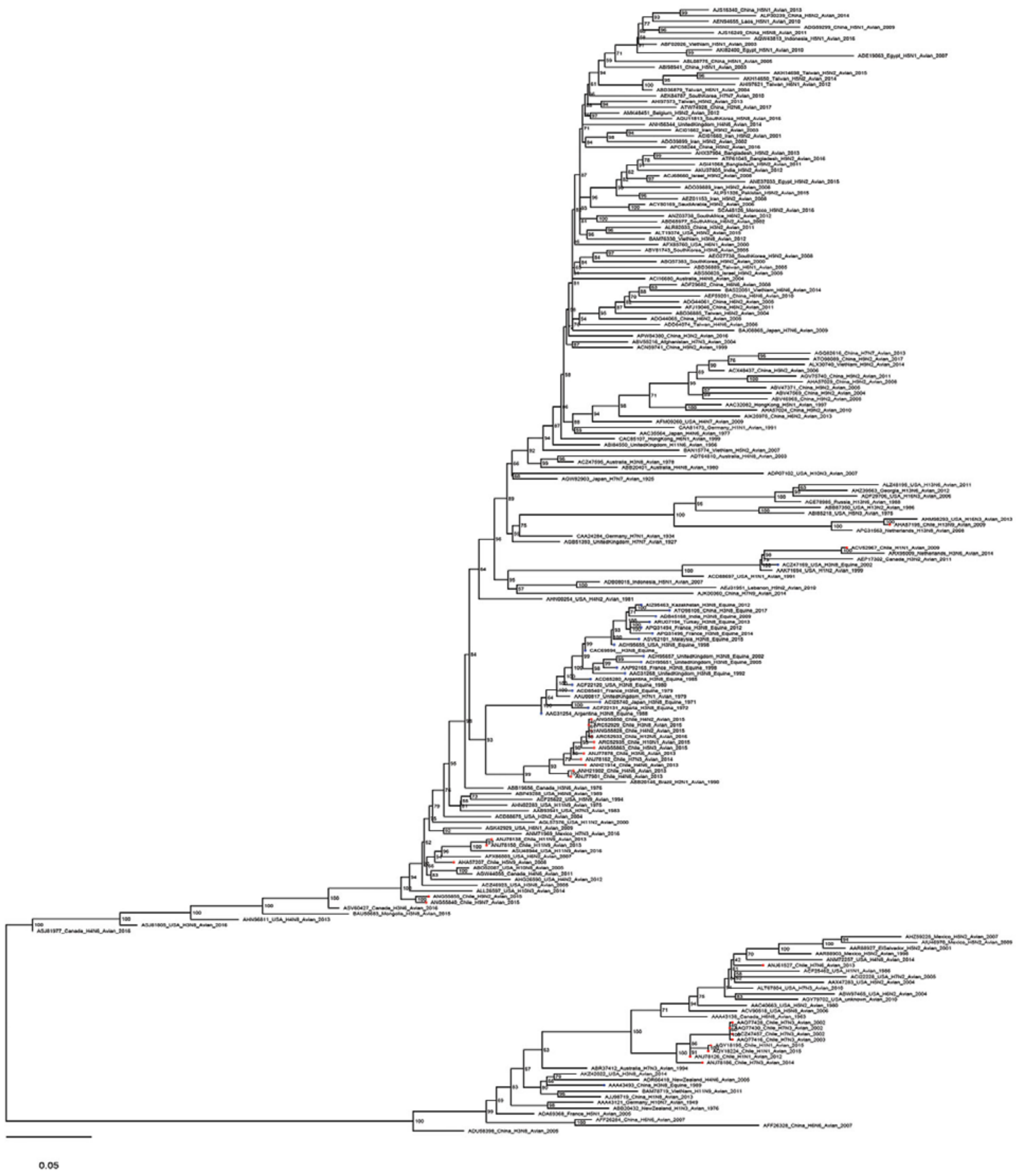

Appendix Figure 3. Maximum-likelihood phylogenetic tree showing the relationship between the H3N8 equine-like influenza virus (EIV) (blue node), avian influenza virus (AIV) from Chile (red node), and AIV samples from other locations for the gene fragment nonstructural protein 1 (NS1). Bootstrap values are labeled next to nodes. Samples from Chile and South America have greater homology to H3N8 EIV in the hemagglutinin 3, nucleoprotein, polymerase acidic, and NS1 gene segments. Scale bar indicates average nucleotide substitutions per site.

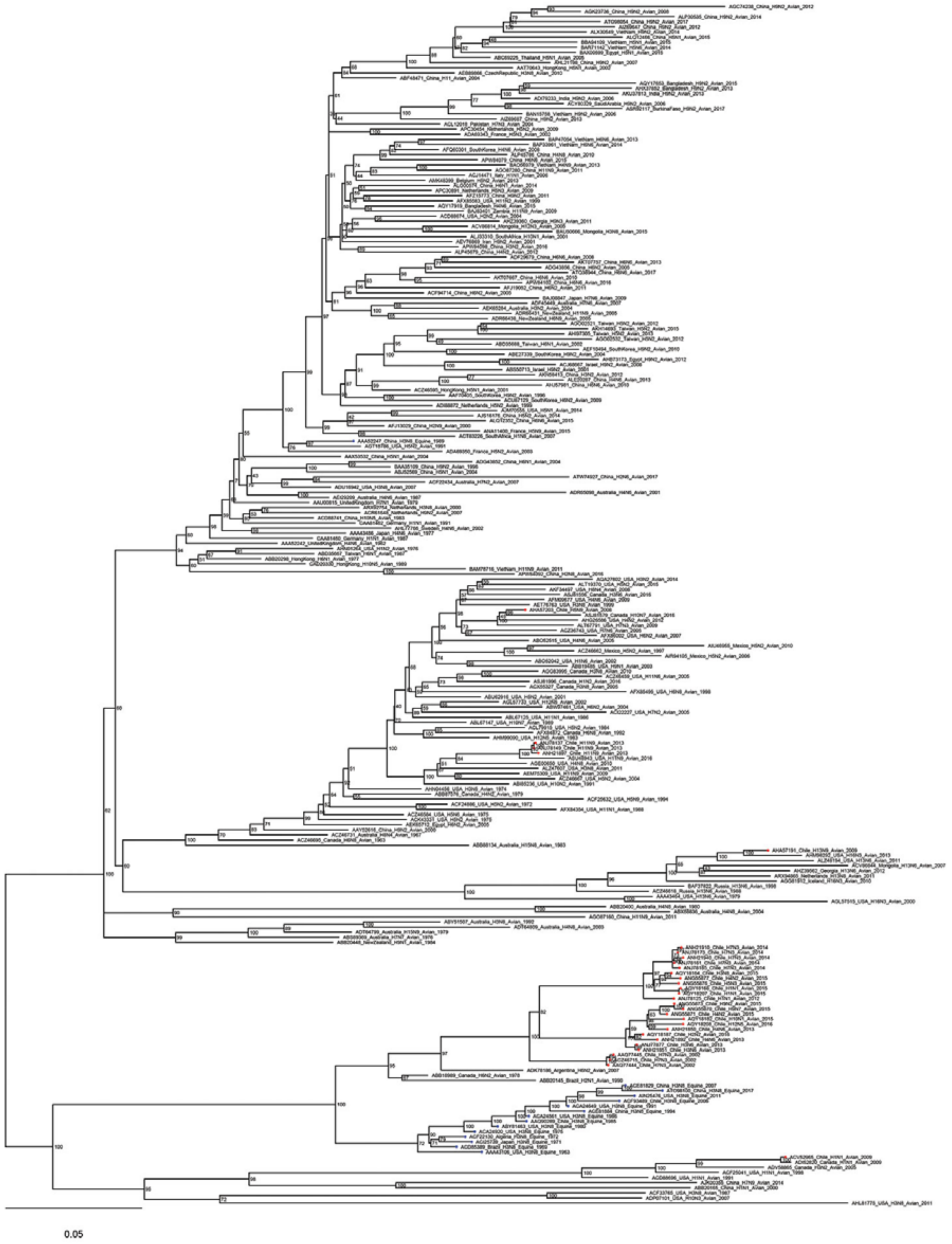

Appendix Figure 4. Maximum-likelihood phylogenetic tree showing the relationship between the H3N8 equine-like influenza virus (EIV) (blue node), avian influenza virus (AIV) from Chile (red node), and AIV samples from other locations samples for the gene fragment nucleoprotein (NP). Bootstrap values are labeled next to nodes. Samples from Chile and South America have greater homology to H3N8 EIV in the hemagglutinin 3, nucleoprotein, polymerase acidic, and NS1 gene segments. Scale bar indicates average nucleotide substitutions per site.

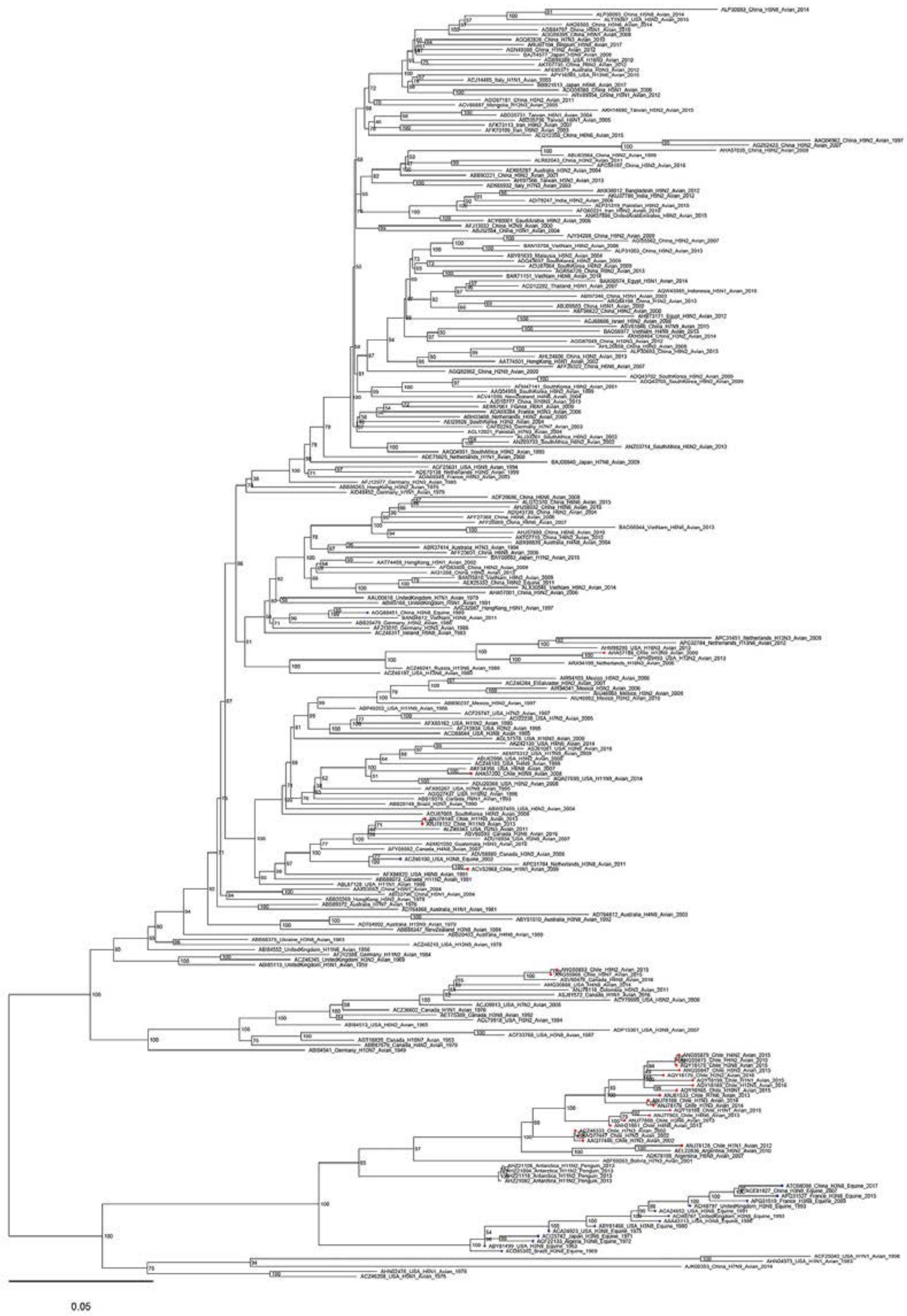

Appendix Figure 5. Maximum-likelihood phylogenetic tree showing the relationship between the H3N8 equine-like influenza virus (EIV) (blue node), avian influenza virus (AIV) from Chile (red node), and AIV samples from other locations for the gene fragment polymerase acidic (PA). Bootstrap values are labeled next to nodes. Samples from Chile and South America have greater homology to H3N8 EIV in the hemagglutinin 3, nucleoprotein, PA, and nonstructural protein 1 gene segments. Scale bar indicates average nucleotide substitutions per site.

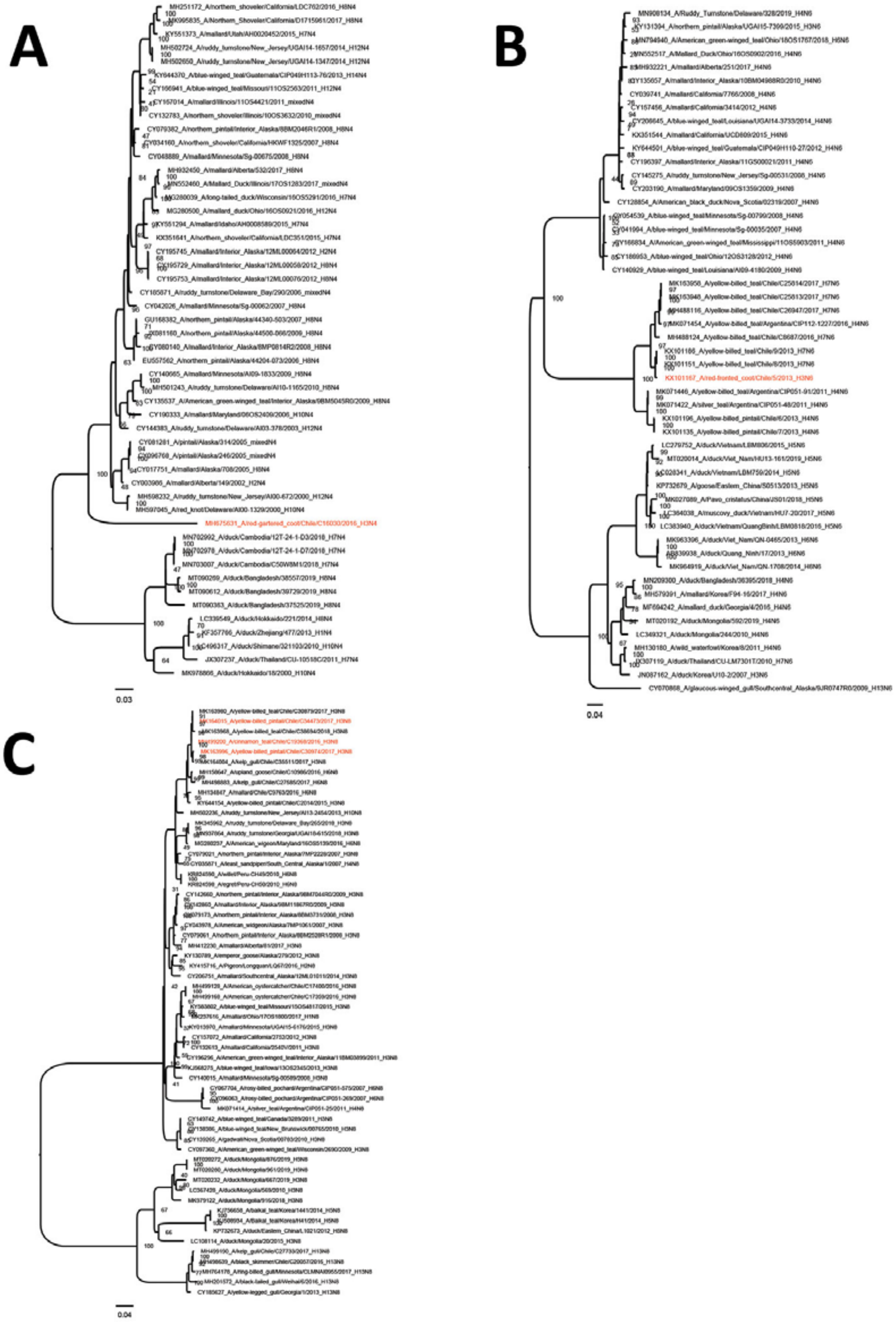

Appendix Figure 6. Maximum-likelihood phylogenetic trees showing the relationship between the neuraminidase (N) gene of H3Nx viruses from Chile used in this study (red) and AIV samples from other locations for this gene fragment. Bootstrap values are labeled next to nodes. Scale bars indicate average nucleotide substitutions per site.

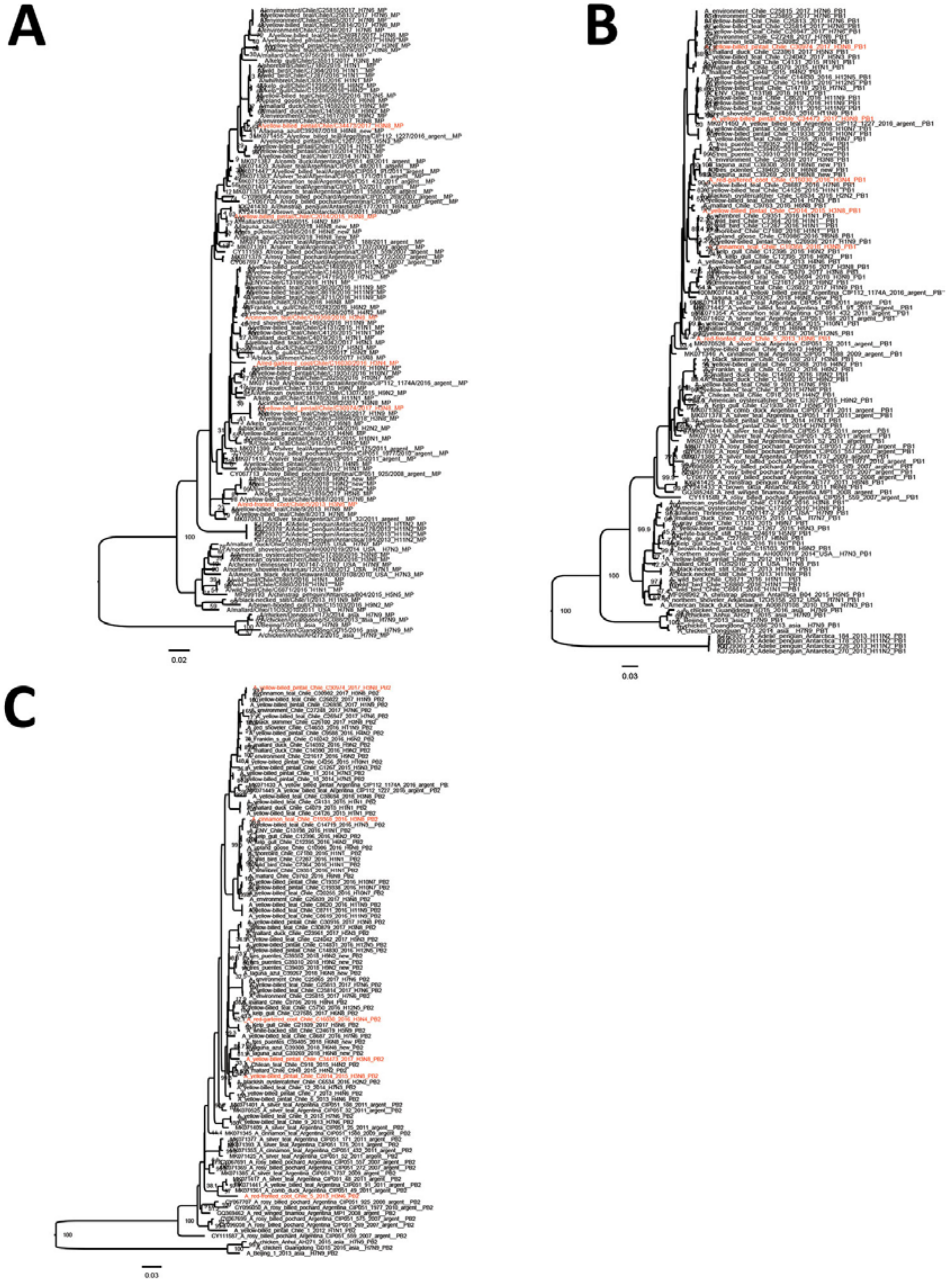

Appendix Figure 7. Maximum-likelihood phylogenetic trees showing the relationship between the MP, PB1, and PB2 of H3Nx viruses from Chile used in this study (red), and AIV samples from other locations for the 3 gene fragments. Bootstrap values are labeled next to nodes. Scale bars indicate average nucleotide substitutions per site. MP, matrix protein; PB1, polymerase basic 1; PB2, polymerase basic 2.

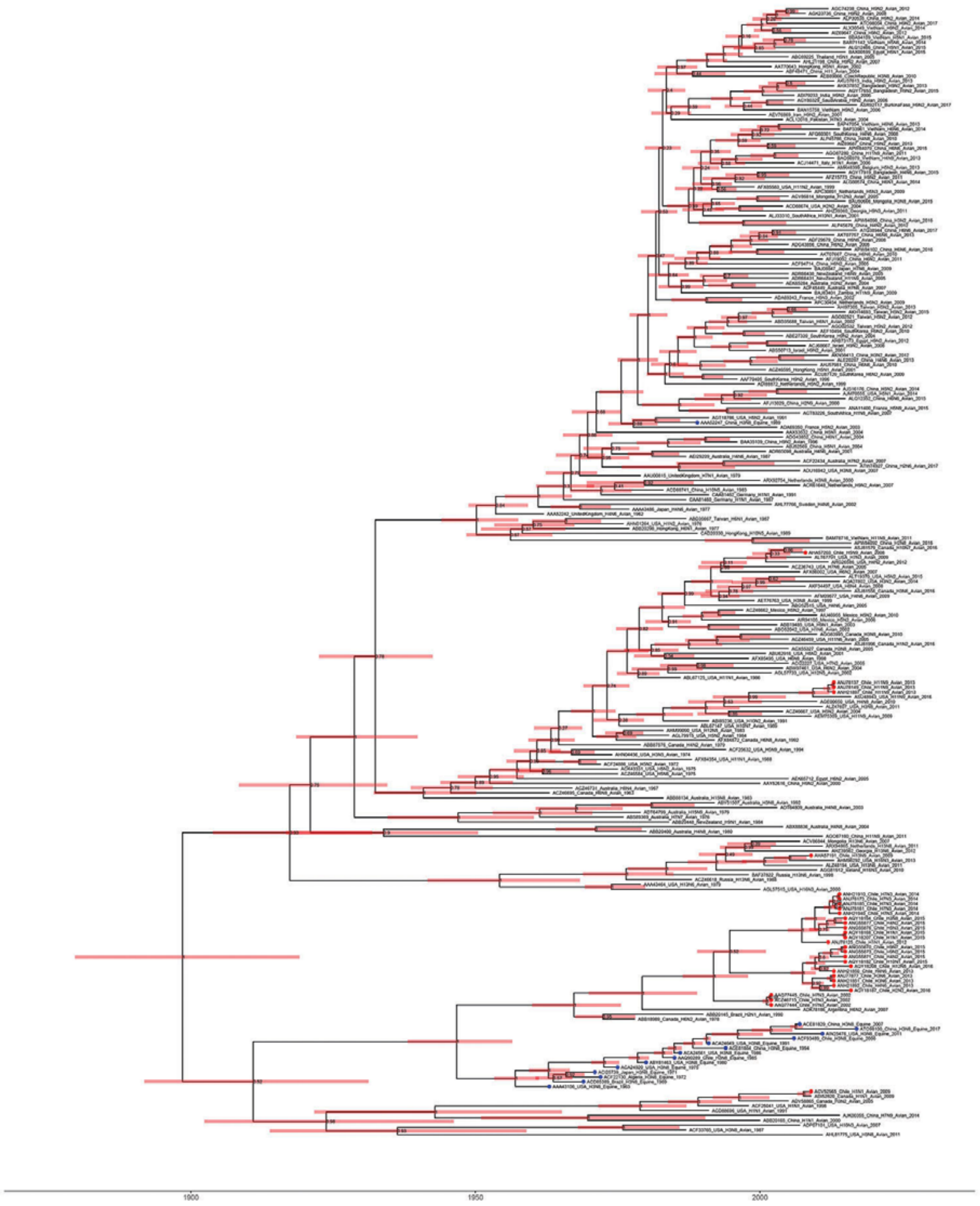

Appendix Figure 8. Maximum clade credibility tree showing the relationship between the H3N8 equine-like influenza virus (EIV) (blue node), avian influenza virus (AIV) from Chile (red node), and AIV samples from other locations for the nucleoprotein (NP) gene fragment. The x-position of the tips represent the year that the virus was sampled. The x-position of the nodes corresponds to the divergence date, with the 95% highest posterior density values shaded in light red. The posterior probability values are labeled next to the nodes. Time scale bar indicates years.

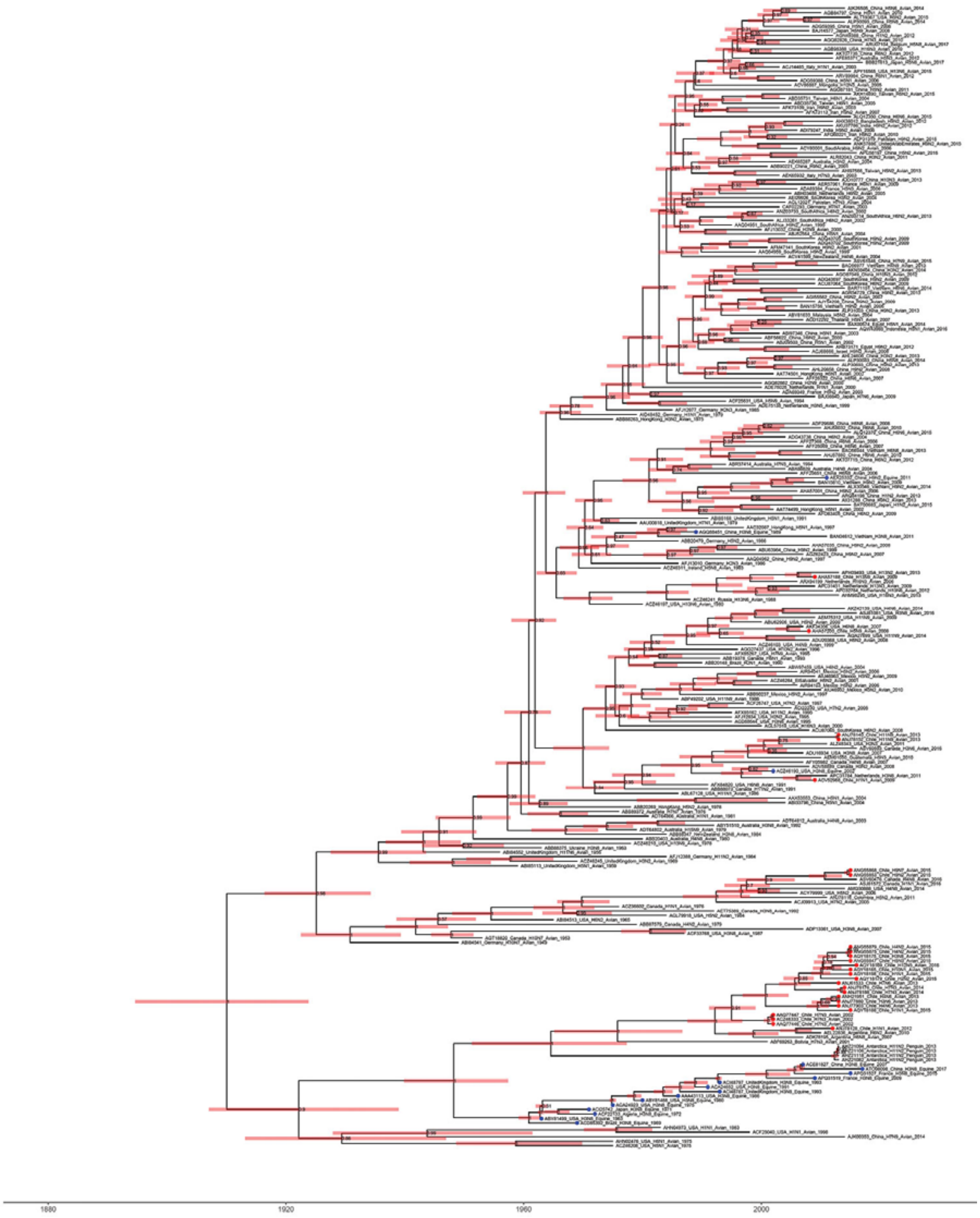

Appendix Figure 9. Maximum clade credibility tree showing the relationship between the H3N8 EIV (blue node), Chilean AIV (red node), and worldwide AIV samples for the polymerase acidic (PA) gene fragment. The x-position of the tips represent the year that the virus was sampled. The x-position of the nodes corresponds to the divergence date, 95% Highest Posterior Density (HDP) values shaded in light red. The posterior probability values are labeled next to the nodes. Time scale bar indicates years.
